# Cavernous Transformation of Portal Vein Secondary to Portal Vein Thrombosis: A Case Report

**DOI:** 10.4021/jocmr775w

**Published:** 2012-01-17

**Authors:** Radhames Ramos, Yoojin Park, Ghulamullah Shazad, Christine A.Garcia, Ronny Cohen

**Affiliations:** a Resident Physician, Woodhull Medical Center, Brooklyn, New York, USA; b Medical Student, School of Medicine, St George’s University, Grenada, West Indies; c Gastroenterology Fellow, Nassau County Medical Center, East Meadow, New York, USA; d Director of Cardiology, Woodhull Medical Center, Brooklyn, New York, NYU School of Medicine, New York, USA

## Abstract

There are few reported cases of cavernous transformation of the portal vein (CTPV) in adults. We present a case of a 58 year-old male who was found to have this complication due to portal vein thrombosis (PVT). A 58-year old African American male with chronic alcohol and tobacco use presented with a 25-day history of weakness, generalized malaise, nausea and vomiting associated with progressively worsening anorexia and weight loss. The patient was admitted for severe anemia in conjunction with abnormal liver function tests and electrolyte abnormalities, and to rule out end stage liver disease or hepatic malignancy. The work-up for anemia showed no significant colon abnormalities, cholecystitis, liver cirrhosis, or liver abnormalities but could not rule out malignancy. An esophageogastroduodenoscopy (EGD) was suspicious for a mass compressing the stomach and small bowel. After further work-up, the hepatic mass has been diagnosed as a cavernous transformation of the portal vein (CTPV), a very rare complication of portal vein thrombosis (PVT). Cavernous Transformation of the Portal Vein (CTPV) is a rare and incurable complication of portal vein thrombosis (PVT) that should be considered as one of the differential diagnoses of a hepatic mass.

**Keywords:**

Cavernous transformation of the portal vein; Portal vein thrombosis; Portal hypertension; Hyperbilirubinemia; Hepatic mass

## Introduction

Cavernous Transformation of the Portal Vein (CTPV) is a rare condition with various etiologies and diverse clinical presentations [1]. It occurs with long-standing portal vein thrombosis (PVT) which causes portal hypertension and an occlusion of the portal vein leading to the development and dilatation of multiple small vessels in and around the re-canalizing main portal vein [2,3]. It has been found to occur commonly in patients with healthy livers with chronic non-cirrhotic and non-tumoral PVT. Yet, the causes of CTPV are unknown [4]. The main clinical presentations are gastroesophageal variceal bleeding and hematologic abnormalities due to the effects of the collateral vessels resulting in an enlarged spleen and the development of porto-systemic collaterals. However, the diagnosis of CTPV is very rarely made on an adult with signs and symptoms of obstructive jaundice. Abdominal ultrasonography, color Doppler ultrasonography, computated tomography angiography (CTA), and magnetic resonance imaging (MRI) are used to confirm its diagnosis. We report a case of a male adult in whom the diagnosis of CTPV was made during the initial workup for a hepatic mass. The symptoms that led us to this rare diagnosis were hyperbilirubinemia and an extrinsic mass compressing on the stomach and the small bowel without any signs of portal hypertension. Although the patient presented with anemia, no sources of bleeding were found, including the presence of esophageal varices.

## Case Report

A 58-year-old African American male with a history of chronic alcohol (a quart of distilled liquor a day for 40 years) and tobacco use (60 pack/years) was brought to the Emergency Department of Woodhull Medical Center by his brother due to progressively worsening fatigue, loss of appetite and a significant weight loss of 40 lb over the last 3 weeks. As per the patient, these symptoms started after his last alcohol consumption 3 weeks ago which was followed by nausea and multiple episodes of vomiting. The vomit was described as clear, with no presence of blood or bile. He denied of any fever, chills, night sweats, hemoptysis, shortness of breath, dizziness or pain. Patient was in mild distress appearing ill. He was alert and oriented to person, place and time.

Vital signs were: blood pressure: 91/50 mmHg, heart rate: 106 beats/minute, respiration rate: 16 breaths/min, temperature: 98.5 ºF, and oxygen saturation: 98% with 2 L nasal cannula.

Physical exam was remarkable for jaundiced sclerae. Abdomen was distended with increased bowel sounds, positive shifting dullness and no tenderness. A healed surgical scar was seen on the medial aspect of the abdomen which was consistent with a history of exploratory laparotomy 9 years ago that the patient could not recall the reason for. Liver was palpated 2 cm under right costal border and spleen was not palpable. Upon rectal exam, guaiac positive stool was demonstrated. Patient had fine intention tremors on both hands.

Laboratory workup showed a marked hypokalemia (1.9 mEq/ml) with magnesium of 1.1 mEq/ml. Calcium was 7.3 mg/dL, but when corrected to albumin, its level was 8.4 mEq/ml. Patient was anemic with the results of hemoglobin 7.0 g/dl and hematocrit 22.5% with a MCV of 118 fL. Coagulation profile showed normal platelets with prothrombin time (PT) of 16.2 sec with INR of 1.94 and activated partial thromboplastin time (aPTT) of 41.4 sec. Liver profile resulted in AST: 87 U/L, ALT: 25 U/L, total bilirubin: 14 mg/dL. An arterial blood gas performed at FiO_2_ of 28% showed: pH: 7.53, PCO2: 38.7 mmHg, PO2: 115 mmHg, O_2_ saturation: 99%, bicarbonate: 32 mEq/L.Urinalysis was consistent with urinary tract infection with positive leukocytes. Chest X-ray and EKG were unremarkable.

Patient was admitted for severe symptomatic anemia, electrolyte imbalance, and to further investigate the abnormal liver profile, and to treat urinary tract infection.

After the electrolyte abnormalities were corrected, patient had abdominal ultrasound and CT scan of abdomen and pelvis with oral contrast. The ultrasound showed gallbladder wall and diffuse circumferential large bowel wall thickening, and a moderate amount ascites which were consistent with cholecystitis and infectious enteritis. Cholecystigraphy (HIDA scan) reported no visualization of gallbladder indicating cystic duct obstruction. Ultrasound with doppler did not show any thrombosis in the infrahepatic area of the portal vein. CT scan of the abdomen and pelvis showed hepatic steatosis, multiple small tortuous vessels in the region of the porta hepatis and variceal veins along common bile duct.

On the second day of admission, the patient developed watery, non-bloody, non-mucous diarrhea. Colonoscopy found a single multi-nodular broad-based polyp in the cecum, moderately congested mucosa in the entire colon, and moderately severe loss of haustral folds in the entire colon. Esophagogastroduodenoscopy (EGD) showed no varices but a moderately severe extrinsic impression was found in the antrum of the stomach and in duodenum. It also showed food in the body of the stomach and a small hiatal hernia. The mucosa was edematous, but there was no abnormal mucosal lesion.

These results were correlated clinically, and after further discussion with the radiology team about the findings on the MRI, the diagnosis of cavernous transformation of the portal vein (CTPV) was made. Gastrointestinal consultants requested for magnetic resonance venography (MRV) of the area, however, radiology concluded that further workup was unnecessary since the diagnosis was already made with the CT scan.

Paracentesis was performed and the ascetic fluid was unremarkable for infection or malignancy. The second hepatic panel showed an improvement with the results of total protein: 5.7 g/dL, albumin: 2.2 g/dL, total bilirubin: 11.1 mg/dL, direct bilirubin: 9.0 mg/L, GGTP: 137 U/L, LD: 211 U/L, AST: 84 U/L, ALT: 25 U/L. The biopsy of the mass near the antrum of the stomach and duodenum was unremarkable. The level of CA-125 marker (54.6 U/mL; normal: < 34.9 U/mL) was slightly elevated but it was minimal to have any significance. Alpha-fetoprotein level was within normal limits. The hepatic viral panel reported negative HBsAg and HBsAb with positive HbcAb and anti-HCV exhibiting anti-HCV genotype 1b.

Over the course of his hospital stay, his leukocytosis trended down and the electrolytes were corrected to normal range. His symptoms of diarrhea subsided and his appetite returned to normal. Patient remained stable, and on the 15th day of his admission, he was transferred to a subacute facility to start a rehabilitation process.

## Discussion

Cavernous transformation of the portal vein (CTPV) is a rare disease in adults. The development and dilatation of multiple small vessels in and around the recanalizing main portal vein leads to long-standing portal vein thrombosis (PVT) [2,3]. Most cases present with signs and symptoms of acute upper gastrointestinal tract bleeding [5], which was not seen in our patient’s presentation. It usually occurs in healthy livers with chronic non-cirrhotic and non-tumoral PVT.

There are several compensatory mechanisms occur when a healthy liver loses about two thirds of its blood supply. First, in the acute stage of PVT, hepatic artery dilates to stabilize the liver by going through an ‘arterial rescue“. Second, within a few days, there would be a rapid development and dilatation of collateral vessels in and around the intrahepatic and extrahepatic biliary tracts, and around gallbladder to bypass the obstruction. This ”venous rescue“ continues in its process and organizes into a cavernous transformation in around 3 - 5 weeks. These compensatory mechanisms can have harmful effects on intestinal circulation.

There is a wide range of presentations in CTPV. Most patients are asymptomatic at presentation. The clinical presentation of CTPV can range from normal biochemical and physical findings to cholestasis and splenomegaly with abnormal hepatic function [4]. In advanced stages, there may be a decrease in liver function due to gradual loss of hepatic mass. In 80% of patients with CVPT, strictures or displacements in biliary ducts and main bile ducts are seen due to the changes of collateral vessels which are located near biliary tracts and gallbladder. This may appear similar to cholangiocellular cancer in Endoscopic Retrograde Cholangiopancreatography (ERCP) and therefore, is called ”Pseudocholangiocarcinoma sign“ (PSCS) [4].

The initial diagnosis of CTPV is seldom made in adults due to various etiologies and clinical presentations. Our patient was diagnosed with CTPV early in his stage when presented with obstructive jaundice, but diagnosis at this stage is rarely made. In addition, mildly increased serum alkaline phosphatase and direct reacting bilirubin levels with an elevation of related enzymes are the result of the compression of biliary tracts by the venous collaterals that run along the extrahepatic biliary tree [6]. All these features were exhibited by our patient.

The diagnosis of CTPV is confirmed by abdominal ultrasonography, color Doppler ultrasonography, CT angiography [2], and MRI [7]. The etiology of CTPV in our patient could not be determined. However, his past history of gallstone and alcoholic pancreatitis can explain the cause of his long-standing portal hypertension. In 1980, there was a case reported in Germany of a patient who had a partial obstruction of bile flow after posttraumatic thrombosis of the portal vein. In this report, the stenosis was caused by varicosis of mural veins consistent with CTPV, a very rare cause of cholestasis [8]. This presentation is very similar to our patient, although there was no history of trauma reported by the patient. However, there is a history of an exploratory laparotomy made nine years before this admission; hence any indications or previous history remains a mystery. In further questioning the patient, he recalled that one of the physicians had mentioned pancreatitis, but no related history could have been obtained from the patient or his family.

It has been demonstrated that the etiology of CTPV determines the outcome, since the management differs with different causes [1]. For our patient, chronic liver disease would be the most logical etiology, but since his history is highly inaccurate, other conditions cannot be excluded.

CTPV is an incurable disease with limited treatment options. Living-related liver transplantation is an effective procedure in treating CTPV. This procedure is very difficult in terms of reconstructing the portal vein [9], and it has only been studied in children and in severe portal hypertension, which our 58-year patient did not have. For the symptomatic biliary obstruction caused by CTPV, there is no consensus to an optimal treatment [10,11]. Endoscopic management of biliary stricture seems to be effective and safe, but such an option was not offered to our patient, since his bilirubin level trended down and he appeared to be a poor candidate to any invasive procedures.
